# Technical validation of an RT-qPCR *in vitro* diagnostic test system for the determination of breast cancer molecular subtypes by quantification of *ERBB2*, *ESR1*, *PGR* and *MKI67* mRNA levels from formalin-fixed paraffin-embedded breast tumor specimens

**DOI:** 10.1186/s12885-016-2476-x

**Published:** 2016-07-07

**Authors:** Mark Laible, Kornelia Schlombs, Katharina Kaiser, Elke Veltrup, Stefanie Herlein, Sotiris Lakis, Robert Stöhr, Sebastian Eidt, Arndt Hartmann, Ralph M. Wirtz, Ugur Sahin

**Affiliations:** BioNTech Diagnostics GmbH, Mainz, Germany; STRATIFYER Molecular Pathology GmbH, Cologne, Germany; Institute of Pathology, University of Erlangen, Erlangen, Germany; Institut für Pathologie am St. Elisabeth-Krankenhaus, Cologne, Germany

**Keywords:** MammaTyper, Analytical validation, Reproducibility, FFPE, RT-qPCR, Breast cancer, *ERBB2*, *ESR1*, *PGR*, *MKI67*

## Abstract

**Background:**

MammaTyper is a novel CE-marked *in vitro* diagnostic RT-qPCR assay which assigns routinely processed breast cancer specimens into the molecular subtypes *Luminal A-like*, *Luminal B-like* (*HER2 positive* or *negative*), *HER2 positive* (*non-luminal*) and *Triple negative* (ductal) according to the mRNA expression of *ERBB2, ESR1, PGR* and *MKI67* and the St Gallen consensus surrogate clinical definition. Until now and regarding formalin-fixed, paraffin-embedded material (FFPE), this has been a task mostly accomplished by immunohistochemistry (IHC). However the discrepancy rates of IHC for the four breast cancer biomarkers are frequently under debate, especially for Ki-67 which carries the highest degree of inter- and even intra-observer variability. Herein we describe a series of studies in FFPE specimens which aim to fully validate the analytical performance of the MammaTyper assay, including the site to site reproducibility of the individual marker measurements.

**Methods:**

Tumor RNA was extracted with the novel RNXtract RNA extraction kit. Synthetic RNA was used to assess the sensitivity of the RNXtract kit. DNA and RNA specific qPCR assays were used so as to determine analyte specificity of RNXtract. For the assessment of limit of blank, limit of detection, analytical measurement range and PCR efficiency of the MammaTyper kit serial dilutions of samples were used. Analytical precision studies of MammaTyper were built around two different real time PCR platforms and involved breast tumor samples belonging to different subtypes analyzed across multiple sites and under various stipulated conditions. The MammaTyper assay robustness was tested against RNA input variations, alternative extraction methods and tumor cell content.

**Results:**

Individual assays were linear up to at least 32.33 and 33.56 Cqs (quantification cycles) for the two qPCR platforms tested. PCR efficiency ranged from 99 to 109 %. In qPCR platform 1, estimates for assay specific inter-site standard deviations (SD) were between 0.14 and 0.20 Cqs accompanied by >94 % concordant single marker assignments for all four markers. In platform 2, the inter-site SD estimates were between 0.40 and 0.66 Cqs while the concordance for single marker assignments was >94 % for all four markers. The agreement reached between the two qPCR systems located in one site was 100 % for *ERBB2*, 96.9 % for *ESR1*, 97.2 % for *PGR* and 98.6 % for *MKI67*. RT-qPCR for individual markers was stable up to a 64-fold dilution for a typical clinical sample. There was no change in assay performance detected at the level of individual markers or subtypes after using different RNA isolation methods. The presence of up to 80 % of surrounding non-tumor tissue including *in situ* carcinoma did not affect the assay output. Sixteen out of 20 RNXtract eluates yielded more than 50 ng/μl of RNA (average RNA output: 233 ng/μl), whereas DNA contamination per sample was restricted to less than 15 ng/μl. Median recovery rate of RNA extraction was 91.0 %.

**Conclusions:**

In this study the performance characteristics of MammaTyper were successfully validated. The various sources of analytical perturbations resulted in negligible variations in individual marker assessments. Therefore, MammaTyper may serve as a technical improvement to current standards for decentralized FFPE-based routine assessment of the commonly used breast cancer biomarkers and for molecular subtyping of breast cancer specimens.

**Electronic supplementary material:**

The online version of this article (doi:10.1186/s12885-016-2476-x) contains supplementary material, which is available to authorized users.

## Background

Despite substantial progress in diagnosis and treatment of breast cancer during the past decades, approximately 15 % of all newly diagnosed breast cancer patients will still die of the disease within the first 5 years [1]. Classic histopathology, immunohistochemistry (IHC), pTNM staging and clinical characteristics have traditionally been used to estimate a patient’s risk of relapse and long-term outcome and provide indications on (neo)adjuvant treatment. In recent years molecular profiling of breast cancer has prompted actions towards more precise stratification of individual patients and more informed treatment decisions [2]. The so called “intrinsic” subtypes (Luminal A, Luminal B, HER2-positive and Basal-like), although not unrelated to long-established breast cancer phenotypes, likely represent separate diseases with distinct underlying biology and clinical characteristics. Accumulating evidence suggests that these molecular entities hold significant information with regard to prognosis, time-point and location of distant metastases and benefit of therapies. The exact determination of these molecular subtypes may enhance the prognostic power of traditional clinicopathological parameters [3].

The prototypic molecular subtypes were identified by microarray technology investigating mRNA expression patterns of 1,753 genes in 84 patient samples [4], 8.000 genes in 122 patient samples [5] or 306 genes in 416 patient samples [6]. Attempts to enable integration of these findings into the routine pathology diagnostic workup led to the development of a 50 gene signature for the determination of the intrinsic subtypes [7] and the transfer of this signature onto a diagnostic platform [8]*.* However, as this approach for subtyping requires specialized instrumentation, it is mainly reserved for diagnostically challenging cases, whereas the current widely adopted standard for breast cancer subtyping is based on a surrogate protein-based classification system for clinical use [9]. This approach has been embraced by the St Gallen International Expert Consensus on the Primary Therapy of Early Breast Cancer since 2011 as a novel paradigm for the classification of patients for therapeutic purposes [2]. These surrogate definitions require a small panel of antibodies against estrogen receptor (ER), progesterone receptor (PR), human epidermal growth factor receptor 2 (HER2) and proliferation antigen Ki-67. Thereby, a manageable, clinically meaningful and continuously updated approximation of the molecular subtypes by immunohistochemistry (IHC) can be achieved. Classification of tumor samples according to these clinico pathological surrogates is carried out by first assessing the binary expression status (positive *versus* negative) of the aforementioned markers. Individual assessments are then used for feeding the classification rules of Table 1, an approach which is conceptually as well as procedurally different than the 50 markers, centroid-based prediction methodology of the Prosigna assay.

**Table 1:** Subtyping algorithm of breast cancer specimens according to St Gallen consensus 2013

*ERBB2*	*ESR1*	*PGR*	*MKI67*	St Gallen 2013 equivalent
pos	pos	pos	pos	*Luminal B-like* (*HER2 positive*)
pos	pos	pos	neg	*Luminal B-like* (*HER2 positive*)
pos	pos	neg	pos	*Luminal B-like* (*HER2 positive*)
pos	pos	neg	neg	*Luminal B-like* (*HER2 positive*)
pos	neg	pos	pos	Not defined
pos	neg	pos	neg	Not defined
pos	neg	neg	pos	*HER2 positive* (*non-luminal*)
pos	neg	neg	neg	*HER2 positive* (*non-luminal*)
neg	pos	pos	pos	*Luminal B-like* (*HER2 negative*)
neg	pos	pos	neg	*Luminal A-like*
neg	pos	neg	pos	*Luminal B-like* (*HER2 negative*)
neg	pos	neg	neg	*Luminal B-like* (*HER2 negative*)
neg	neg	pos	pos	Not defined
neg	neg	pos	neg	Not defined
neg	neg	neg	pos	*Triple negative* (*ductal*)
neg	neg	neg	neg	*Triple negative* (*ductal*)

In recent years, growing concerns over the reproducibility of routine assessment of breast cancer biomarkers by IHC with reported discordance rates of up to 20 % for ER and HER2, have motivated the development of detailed guidelines to improve the accuracy of testing [10–13]. While the average discrepancy rate in defined clinical settings for ER, PR and HER2 is frequently under debate, it is beyond any doubt that among all four breast cancer biomarkers, Ki-67 carries the highest degree of inter- and even intra-observer variability which makes scoring particularly hard to reproduce even between experienced pathologists [14, 15]. Reported intra-observer Kappa values of as low as 0.00–0.35 illustrate an alarming variability of the current Ki-67 scoring methods [15]. Although no study has yet addressed the relation between single marker discrepancies and the rate of misclassification of breast tumors into molecular subtypes, it is probable that significant uncertainty exists particularly in the critical distinction between Luminal A- and B-like tumors, due to multiplying error probabilities of individual markers. Recent efforts to improve the concordance of Ki-67 scoring have demonstrated that agreement between pathologists using a specific scoring method and following training can lead to a significant improvement of scoring concordance. However, these standardized methods are not yet adopted widely and also lack clinical validation [16, 17].

The limitations of current methods for routine molecular subtyping of breast cancer highlight the need for more reliable, well standardized and less subjective assays. However, it is also important that these assays can be performed in a decentralized environment, where close collaboration between pathologists and clinicians and faster turnaround are beneficial for both patients and medical practitioners. Reverse-transcription quantitative real time PCR (RT-qPCR) has been previously considered a reasonable alternative to IHC due to several competing advantages; it is quantitative, it is not affected by inter-observer variability, interpretation of results is straightforward and the technique can be performed locally in a standardized and automated manner largely irrespective of sample size [18–20].

The MammaTyper gene expression assay is a CE-marked *in vitro* molecular diagnostic test which measures the mRNA expression levels of the four genes *ERBB2, ESR1*, *PGR* and *MKI67* in surgical breast cancer samples and pre-operative biopsies to assign a tumor to a molecular subtype (*Luminal A-like*, *Luminal B-like* (HER2 positive or negative), *HER2 positive* (non-luminal) and *Triple negative* (ductal)). The quantitative format of biomarker detection has the potential to be used for the prediction of response to systemic treatments [21, 22]. The assay is based on RT-qPCR of total RNA extracted from formalin-fixed paraffin-embedded (FFPE) material and can be run locally on widely accessible qPCR instruments. The workflow includes a novel standardized nucleic acid extraction kit (RNXtract RNA Extraction Kit, BioNTech Diagnostics GmbH, Mainz) for the isolation of high-quality tumor RNA.

Results from a recent clinical validation study in 769 patients from the FinHER clinical trial population showed that subtyping by MammaTyper results in a better prediction of patient outcome than when subtyping was based on local IHC data (Wirtz et al. submitted). Accordingly, the test result is prognostic for the patients’ risk for distant metastases and overall survival and supports the prediction of benefit from the addition of taxane to adjuvant chemo-endocrine therapy. The clinical performance of the MammaTyper assay is currently interrogated in multiple additional prospective/retrospective settings so as to meet the high standards for clinical validity required for diagnostic applications tested on archived specimens [23].

Apart from clinical validation, recent guidelines highlight the importance of formal testing procedures for estimating a diagnostic assay’s analytical performance before it is accepted for clinical use [24]. Herein we undertook a rigorous analytical validation of the RNXtract kit and the MammaTyper assays accounting for multiple factors of the analytical process from tumor RNA isolation and the assessment of each single biomarker to the algorithmic determination of breast cancer molecular subtypes. The design of the studies has been optimized to make best use of established guidelines such as the evaluation of precision of quantitative measurement methods (EP5-A3) issued by the Clinical and Laboratory Standards Institute (CLSI) and the Minimum Information for Publication of Quantitative Real-Time PCR Experiments (MIQE) guidelines [25, 26]. Moreover, two of the testing sites were independent molecular pathology laboratories.

## Methods

### Sample selection and assay description

RNA for each sample was extracted from a single non-macrodissected 10 μm-thick FFPE section with the RNXtract kit (BioNTech Diagnostics GmbH). Pathologic review of a representative H&E-stained slide (biopsy or resection specimen) ensured the presence of sufficient tumor tissue with at least 20 % tumor cell content. A description of basic clinical and pathological characteristics of the samples used herein may be found in Additional file 1A. The RNXtract RNA extraction kit employs germanium-coated paramagnetic particle technology in order to segregate RNA from FFPE material with preference over DNA. The procedure begins with paraffin melting and buffer-controlled tissue lysis, followed by proteinase K digestion. Thereafter, lysates are incubated with magnetic beads under optimized buffer conditions which favour binding of RNA molecules. Contaminants are then progressively removed through sequential washing steps, during which paramagnetic particles with attached nucleic acids are kept magnetized, while the supernatant is removed. At the final step, RNA is released from the magnetic beads into 100 μl of elution buffer.

With MammaTyper reverse transcription of RNA and amplification of cDNA take place successively in one reaction mix, which contains all the necessary enzymes and hydrolysis primer/probe sets specific for the target sequences of interest. For the various analytical performance experiments forty cycles of nucleic acid amplification were applied and the quantification cycle (Cq) values of the target and reference genes were estimated as the median of triplicate measurements per RT-qPCR run. Median Cq values of the triplicate measurements (from now on simply Cq) for each of the 4 different genes of interest (GOI) were normalized against the mean expression of the two reference genes (REF) and presented as ΔΔCq values relative to the positive control [27]. The final values were generated by subtracting ΔΔCq from the total number of cycles so that test results are positively correlated, a format that facilitates interpretation for clinical decision making:

$$ 40-\varDelta \varDelta \mathrm{C}\mathrm{q}{\left(\mathrm{G}\mathrm{O}\mathrm{I}\right)}_{\mathrm{S}} = 40-\left(\left(\mathrm{C}\mathrm{q}{\left[\mathrm{G}\mathrm{O}\mathrm{I}\right]}_{\mathrm{sample}\ \hbox{--}}\mathrm{meanCq}{\left[\mathrm{R}\mathrm{E}\mathrm{F}\right]}_{\mathrm{sample}}\right)\hbox{--}\ \left(\mathrm{C}\mathrm{q}{\left[\mathrm{G}\mathrm{O}\mathrm{I}\right]}_{\mathrm{pc}}\hbox{--}\ \mathrm{meanCq}{\left[\mathrm{R}\mathrm{E}\mathrm{F}\right]}_{\mathrm{pc}}\right)\right) $$

The test output comprises the normalized raw data of individual biomarkers and the molecular subtype of breast cancer, according to the algorithm depicted in Table 1. Each sample is assigned into *Luminal A-like*, *Luminal B-like* (HER2 positive or HER2 negative), *HER2 positive* (non-luminal) and *Triple negative* subtypes, after dichotomizing continuous RNA output values per marker into “Positive” and “Negative” results according to clinically established and qPCR device-specific cut-offs (Additional file 1B). Assay controls are included to ensure that specimens and RT-qPCR runs satisfy pre-specified quality thresholds. These controls comprise two internal reference genes (REF) for assessment of sample validity and normalization (*B2M*, *CALM2*) as well as one positive (PC) and one negative (no-template control; NTC) external control for qualification of the RT-qPCR process (Additional file 1C). The aforementioned cut-off values for *ERBB2, ESR1* and *PGR* were established in a cohort of 135 breast cancer patients, based on the highest concordance achieved between relative RNA amplification and corresponding protein expression by high-quality IHC as the gold standard.

Due to the uncertainty of Ki-67 IHC data the *MKI67* cut-off of the assay under development (Stratifyer Molecular Pathology GmbH) was set at the 3^rd^ quartile of the normally distributed *MKI67* expression data from 90 FFPE breast cancer tumor samples. These had been previously analysed in the context of a clinical outcome prediction study [28]. The cut-off was then transferred from the assay under development to the MammaTyper IVD by parallel measurement of 135 clinical breast cancer samples and matching of cut-offs on this sample set.

### Analytical specificity and analytical sensitivity

It is common that DNase I digestion is included as an extra step during the process of RNA quantification in order to eliminate genomic DNA (gDNA) contamination and increase specificity of the extraction-amplification sequence. In order to test the analytical specificity of the RNXtract method, eluates were prepared from 20 breast cancer samples and equally divided in two aliquots for the estimation of the amount of RNA or DNA with the standard curve method. For this purpose, in aliquot No 1, *B2M* RNA transcripts were measured by RT-qPCR using serial dilutions of the MCF7 cell-line total RNA (BioCat) as standard curve, while in aliquot No 2 qPCR was applied using a commercial gDNA quantification assay for qPCR (primer design) and serial dilutions of a commercial gDNA as standard curve. All experiments were performed on a Roche LightCycler 480 II qPCR instrument in triplicates. For assessing the effect of potential contamination by gDNA on the MammaTyper result, RNA eluates from three different samples were compared under varying conditions; according to standard methodology (non-digested samples), digested with DNase I (Ambion) and treated like DNase I digested samples, where DNase I was exchanged for water. Samples were processed in one run with positive and negative controls.

For assessing the sensitivity of extraction, 3 FFPE samples (1 × 10 μm) were spiked with commercial 4 μl unique internal control RNA (Int-RNA) (primer design) at the initial extraction step so as to determine the recovery rate. The obtained Cq values were compared against a standard curve of Int-RNA serially diluted in RNXtract elution buffer. Since the original concentration of the Int-RNA was not known, extraction efficiency was estimated relative to that of the control sample (representing 100 % input). In addition, the extraction efficiency was validated with respect to variations in the range of input material. Two resection specimens and 6 standard core tumor biopsies (10 μm × 1.5 mm × 20 mm) were serially cut and 10 samples were prepared that differed by specimen type (resection versus biopsy), thickness of paraffin curls (5 μm versus 10 μm) and number of input curls (1, 2 or 3). The RNA concentration of the eluates was quantified with calibration to a standard curve as described above for specificity.

The analytical sensitivity of the RT-qPCR was validated under conditions of reduced RNA template and with respect to the test detection capacity according to the standard methods provided in the EP17-A guideline issued by the Clinical and Laboratory Standards Institute (CLSI) [29]. Serially diluted samples from 3 RNA eluates corresponding to different subtypes and RNA eluates from six tumor biopsies were analyzed and the acceptable range of *B2M* and *CALM2* REF genes was used as a quality control of sample validity according to the instructions for use. For further characterizing the analytical performance of MammaTyper the following formal validation metrics were used. The LoB (limit of blank) i.e., the highest measurement that is likely to be observed for a blank sample, was determined separately for each assay mix and for each qPCR system on 20 replicates of NTC samples containing water (120 reactions in total). Assuming a Gaussian distribution of the Cq values, the LoB, was set at a mean Cq value of 40, observed 95 % of times for each assay mix. The LoD (limit of detection), defined as the smallest analyte concentration likely to be consistently distinguished from the LoB and at which detection is feasible with a certain degree of certainty was herein perceived as the amount of total sample RNA (positive for all targets) at which the Cq value was below the LoB with a probability of 95 %. The LoQ (limit of quantification) was here defined as the amount of target RNA for each marker producing a Cq value which was (a) lower or equal to the Cq determined for the LoD, and (b) displayed a distance to the regression curve of the dilution series lower than 0.7 Cqs for each dilution of the clinical sample or the IVT-RNA (*in-vitro* transcribed RNA). The distance value reflects previously reported PCR replicate variation range of 0.5 to 1.0 Cq [30]. The analytical measurement range for MammaTyper assay was determined as the Cq range over which the MammaTyper shows a linear signal (distance from regression curve ≤0.7 Cq) beginning at the LoD as the lowest input value. For the LightCycler the analytical measurement range is limited by the fix baseline settings (3–15).

A master sample prepared by pooling three eluates from a single patient block (3 FFPE sections per extraction) was measured with spectrophotometry (NanoDrop; Thermo Scientific) and serially diluted in carrier RNA (10 ng/μl). Dilution curves were analyzed with all three assay mixes on both amplification systems. The same procedure was carried out for MammaTyper IVT-RNA. The numbers of molecules corresponding to the highest concentration of the IVT-RNA were calculated online with a web-based molecular weight calculator (http://www.currentprotocols.com/WileyCDA/CurPro3Tool/toolId-8.html). The number of molecules in the master sample was extrapolated from the respective linear function of the regression curves of the IVT-RNA dilution series for each marker and reference gene. Concentrations corresponding to Cq values outside the analytical measurement range (as defined by IFU-based instrument settings) were excluded from the analysis.

### Robustness

In the context of the robustness study the extraction and RT-qPCR workflows were varied at various steps as described in detail in Additional file 1D. RNA was extracted from one FFPE clinical sample and was quantified using the standard curve method. The stability of RT-qPCR against pre-specified fluctuations of protocol parameters was determined as the concordance between subtypes generated under the varying conditions and of subtypes assigned under the standard protocol to 4 clinical breast cancer cases.

### Pre-analytical processing concordance and tumor cell content study

The following studies were designed in order to verify that variability in pre-analytical processing steps, including different methods for RNA extraction or the use of macro-dissected versus full-face tissue sections, do not substantially interfere with the stability of the RT-qPCR-based MammaTyper breast cancer subtypes. For this purpose RNA was extracted from 8 clinical FFPE samples with RNXtract and two other commercially available kits for extraction of RNA from FFPE tissues. Eluates corresponding to the different extraction methods were analyzed within the same RT-qPCR run.

Pathologically confirmed non-invasive tumor tissue including normal breast lobules or ductal carcinoma in situ (DCIS) is commonly part of full-face tumor sections used for RNA extraction and is generally considered a source of errors in gene-expression quantification by RT-qPCR. To assess the possibility that contamination by non-invasive tumor may affect the MammaTyper results, 9 clinical breast cancer cases were selected in order to represent samples with low tumor cell content (10–50 %) which were then enriched up to 80 % upon careful macrodissection. The differences between macrodissected and non-macrodissected samples were recorded as disagreement affecting the status of individual markers. By intention, all cases included DCIS of variable extent (10-70 %) adjacent to tumor.

### Analytical precision and reproducibility

For determining the reproducibility of MammaTyper, two studies were performed on the two compatible qPCR platforms, whereby the experiments were designed according to hierarchy of investigated factors (Study 1: Instrument/site – days – runs – replicates within runs; Study 2: Instrument/site – extraction – run (=days) – replicates within runs). The first study was conducted on four LightCycler 480 instrument II (Roche) devices utilizing pooled RNA extracted from breast tumors. The outset of the second study, conducted on three Versant kPCR Cyclers (Siemens) and one LightCycler 480 instrument II (Roche), was FFPE breast tissue sections and the aim was to investigate pre-analytical factors and variations between qPCR instruments from different suppliers. All operators participated in familiarization runs and were blinded to any characteristic of the test samples which might create anticipation for a specific output. Each sample was tested in triplicates during each run along with the specified positive and negative controls. One, 10 μm-thick tissue section was input for RNA extraction irrespective of tumor surface or tumor cellular content. The results of the two studies have been combined and arranged by qPCR system for presentation purposes.

### Design of study 1

This comparative three-site study was conducted with the MammaTyper by two operators on four LightCycler 480 II (Roche) devices to assess analytical performance as presented in Table 2. The four instruments were in place at the National Centre for Tumor Diseases in Heidelberg, the BioNTech Diagnostics GmbH in Mainz and the University Medical Center of the Johannes Gutenberg University Mainz. Reference RNA (RNA from several cancer cell lines) (Agilent) and RNA pools from seven breast tumor samples were generated from commercially available FFPE specimens for testing at each site. The samples were selected to include the entire range of the clinically anticipated expression levels of the target genes, including values adjacent to the cut-offs. Each sample was processed during six RT-qPCR cycles, run on consecutive days at sites 1 and 2 but not at site 3. The procedure was performed by the first operator at site 1 and by the second operator at the two other sites while at site 2 two different qPCR devices were used.

**Table 2:** Overview of precision studies

	Numbers	
Study variable	Study 1	Study 2
Samples	8	16
Intra-run replicates	3	3
Runs per device	6	9
Runs per day	2	1
Reagent lots per site	1	3/2^a^
Operators per site	1	1
Runs per site	6/12^b^	9/27^c^
Sites	3	3
Different qPCR instruments	4	4^d^
Operators	2	3
Total tests per device	48	144/288^a^
Total tests	192	720

^a^Three different lots of MammaTyper and two lots of RNXtract were used at BioNTech Diagnostics to account for inter-lot precision

^b^Including two devices at BioNTech Diagnostics

^c^Including inter-lot precision and 1 Roche qPCR system at BioNTech Diagnostics

^d^Including 1 Roche qPCR system at BioNTech Diagnostics in order to address variations caused by different instruments

### Design of study 2

This comparative three-site study used replicate breast tumor tissue specimens from the same FFPE block for testing with the MammaTyper (Table 2). A 16-member panel of breast cancer samples (15 ductal carcinomas and 1 lobular carcinoma) obtained from commercial vendors except for one clinical sample and comprising all different tumor subtypes were analyzed. All tissue specimens were sectioned at BioNTech Diagnostics GmbH. The first and last section were analysed with MammaTyper at site 1 and showed nearly identical marker expression. After rearranging, sections were shipped to the other testing sites (Stratifyer Molecular Pathology GmbH, Cologne and Institute of Pathology, University of Erlangen). Each tissue sample was processed during three, 4-day cycles, starting with RNA extraction and aliquoting of eluates (day 1) followed by three RT-qPCR runs on consecutive days (day 2–4). The procedure was performed by one operator per site using a single instrument (Versant kPCR Cycler, Siemens), a single lot of RNXtract and a single lot of the MammaTyper Assay. At BioNTech Diagnostics six additional cycles were performed by the same operator, two with different MammaTyper lots, one with an alternative RNXtract lot and three on a LightCycler 480 II (Roche) qPCR device to account for related effects on precision.

Intra-run precision was estimated based on the variance of triplicate measurements. All calculations for the precision studies were carried out based on CLSI guideline EP 05-A3 [25] using a random effects model II ANOVA via PROC mixed in SAS Version 9.2.

## Results

### Analytical specificity and analytical sensitivity

Quantification of nucleic acids extracted from 20 FFPE samples showed preferential extraction of RNA with a mean concentration of 232.8 ng/μl (95 % CI: 132.8-332.8 ng/μl). By contrast, DNA levels remained consistently below 15 ng/μl with a mean concentration of 5.5 ng/μl (95 % CI: 3.7-7.3 ng/μl) (Additional file 2A).

For the amplification assay, specificity was determined by comparing the status of individual markers as well as subtypes in 3 pairs of DNase I-treated and non-treated samples. Under these varying conditions no difference was observed between DNA-free samples and samples potentially contaminated by DNA. Subtype and single marker concordance was 100 % and no detectable amplification was noticed for negative controls (Cq = 40) (Additional file 2B).

**Fig. 1:**
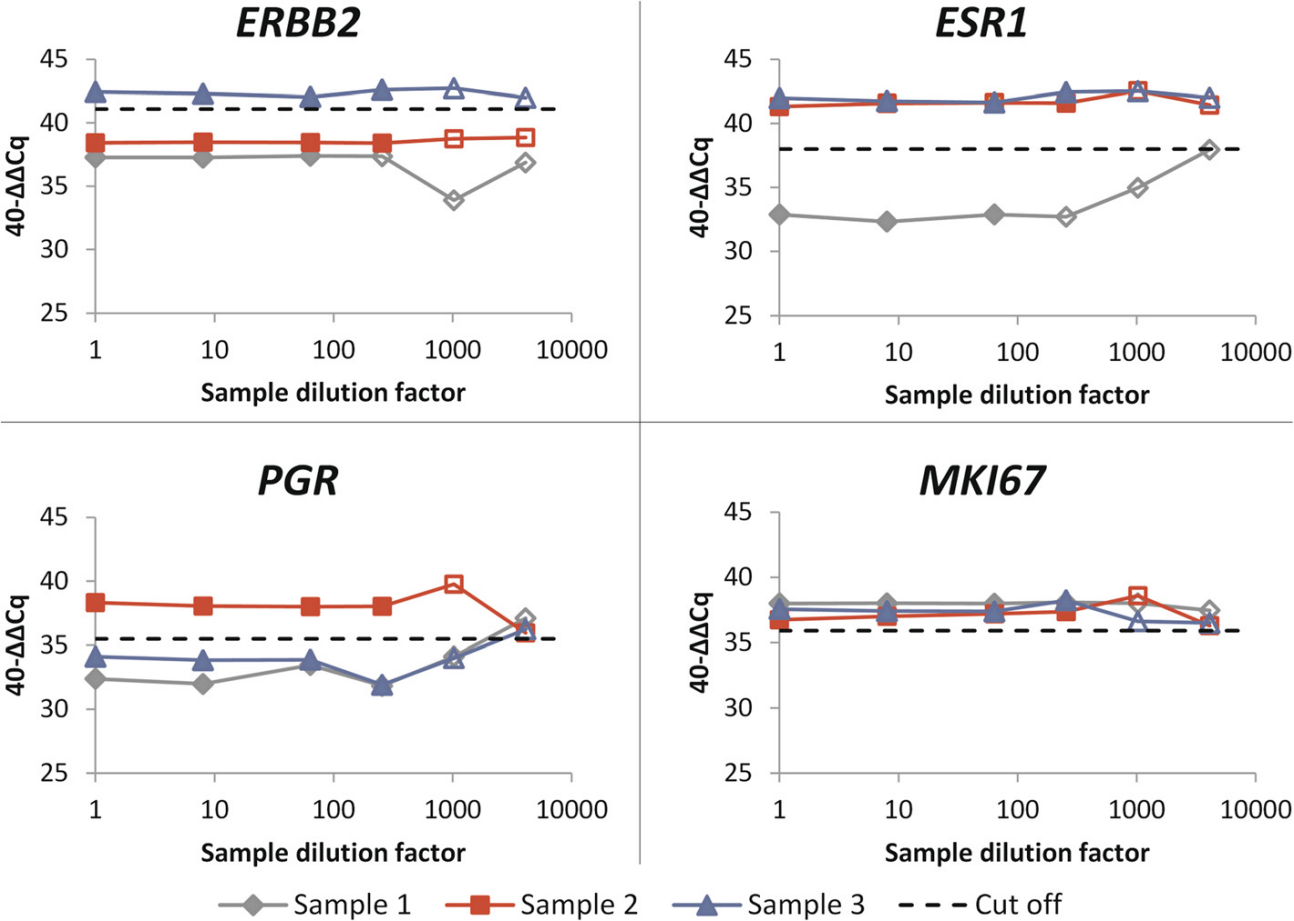
Performance of MammaTyper assay on serial dilutions of 3 breast cancer resection specimens. Filled symbols: Valid measurements. Unfilled symbols: Invalid measurements

The recovery rate of the 3 samples spiked with the unique internal control was 73 %, 91 % and 99 %. Further, RNA isolation was optimal for all variations of the input material sensitivity experiment and remained consistently above 10 ng/μl. Accordingly, RNA-rich eluates were obtained even when samples were reduced to single core needle biopsy or single 5 μm-thick full-face slices. Amplification sensitivity was assessed on three serially diluted samples (*Triple negative*, *Luminal B-like* (HER2 negative) and *HER2 positive* (non-luminal)) and on RNA extracted from six standard core needle biopsies. All marker results of the diluted samples were in agreement with the results of the non-diluted specimen up to the limit of sample validity as defined by the pre-specified *B2M* and *CALM2* Cq thresholds. Further, as shown in Fig. 1, some of the individual marker assignments remained stable up to a dilution factor of 256. All six biopsy samples were valid according to assay specifications resulting into efficient subtyping of the respective tumors.

### Pre-analytical parameters study and robustness

**Fig. 2:**
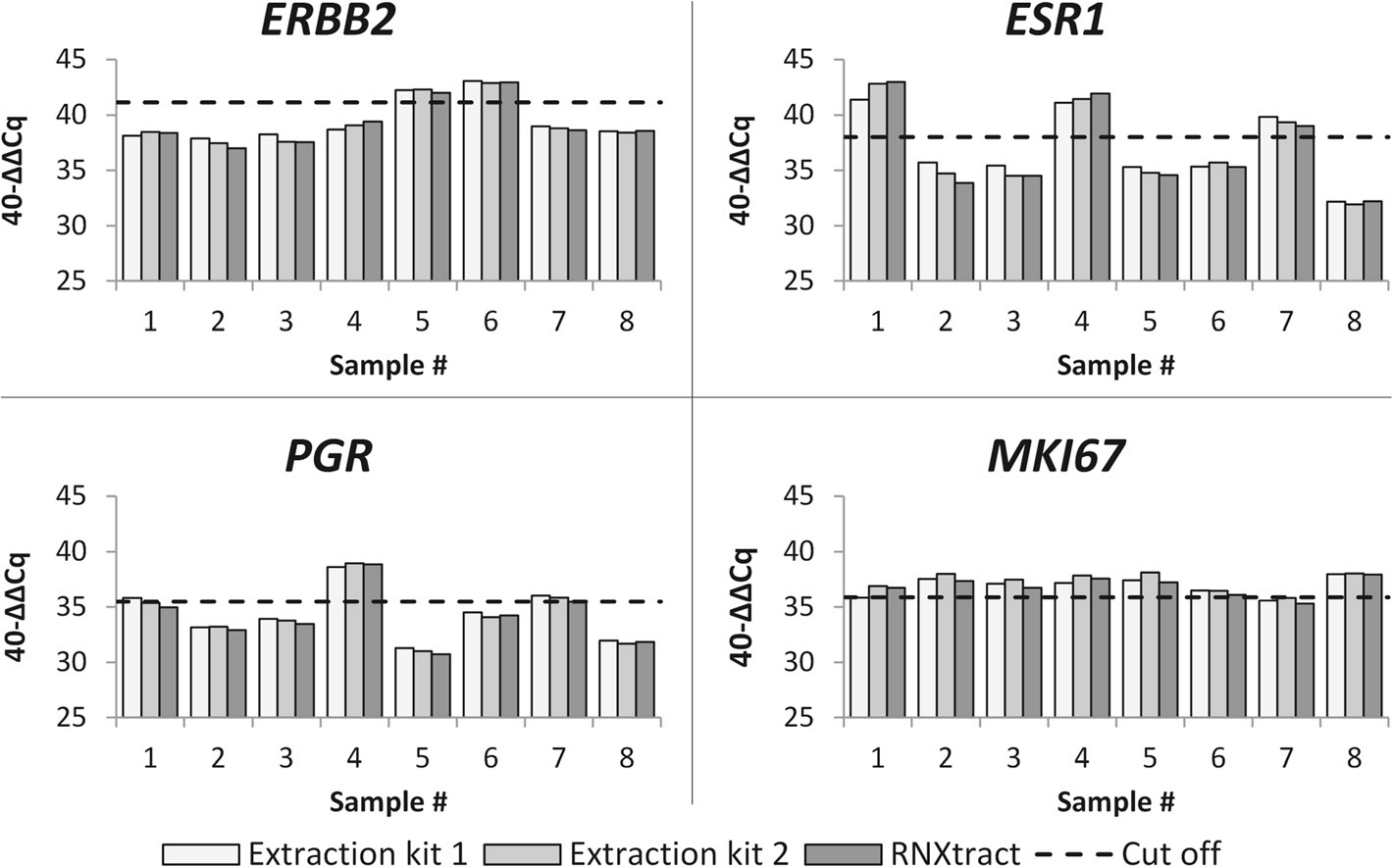
Effect of different commercial extraction kits on the performance of MammaTyper relative quantification of target genes, demonstrated at 8 samples

Three 10 μm-thick sections were generated from each one of eight FFPE tissue specimens and manually processed with RNXtract and two additional commercially available kits for extraction of RNA from FFPE tissue in order to estimate the effect of different isolation methods on MammaTyper assay performance. Among the binary results resulting from 96 measurements (valid specimens: 100 %) there were three discrepant marker classifications involving *PGR* and *MKI67* 40-ΔΔCq values which were close to the respective cut-offs (Fig. 2).

**Fig. 3:**
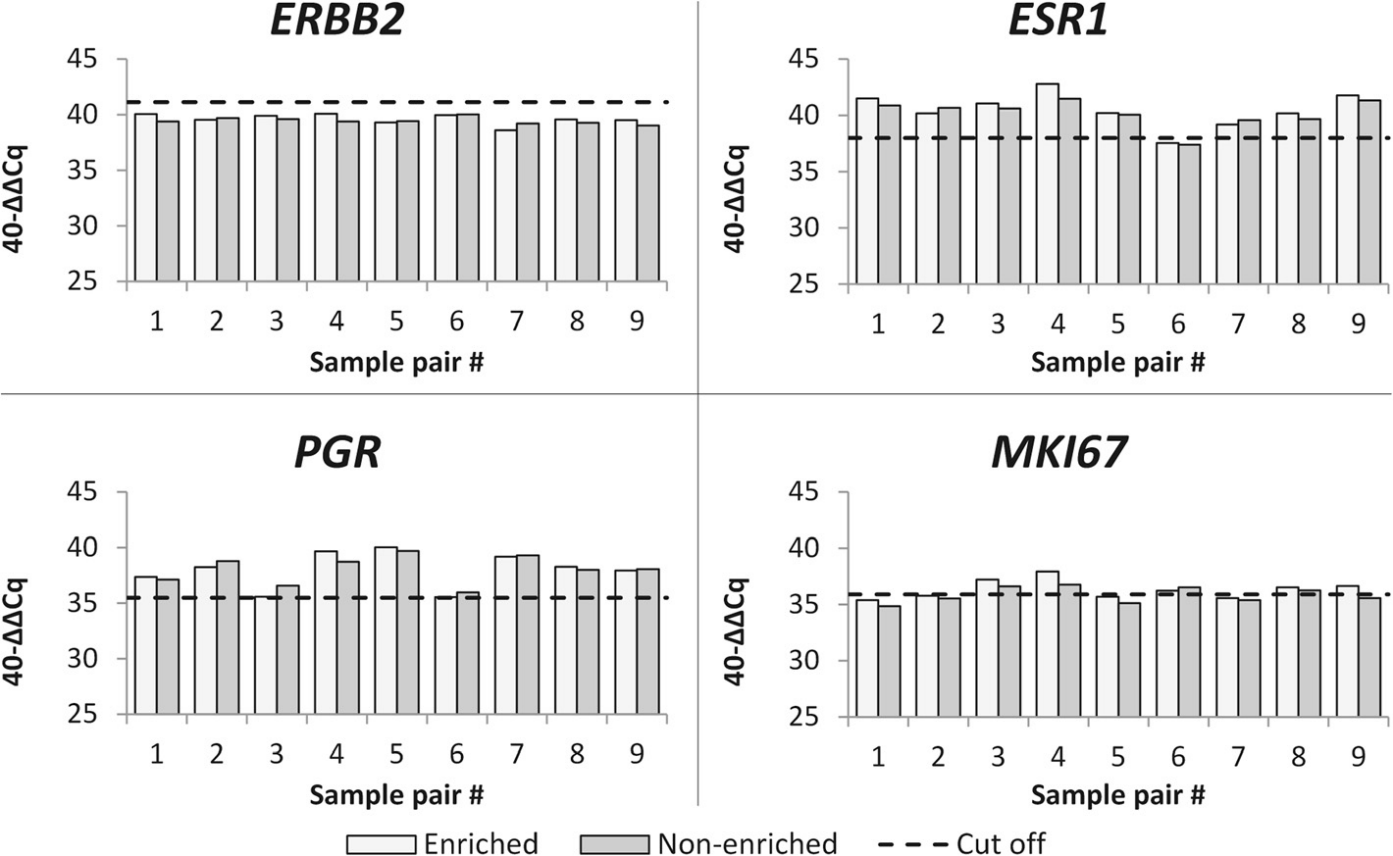
Effect of tumor cell content on the accuracy of MammaTyper relative gene expression, demonstrated at 9 samples

For estimating the effect of tumor cellularity on the accuracy of MammaTyper output, pairs of dissected and non-dissected samples were compared with respect to relative gene expression and binary marker status. One sample did not satisfy validity criteria and was excluded from further calculations. As shown in Fig. 3, 40-ΔΔCq differences across the tested pairs remained very low for all markers, with an average difference of less than 0.47 Cq. Concordance was 100 % for *ERBB2*, *ESR1*, *PGR* whereas for *MKI67*, one case containing 10 % tumor cells was negative in the non-macrodissected sample. Macro-dissection of FFPE tissue sections for gene-expression analysis with the MammaTyper assay may be spared for samples containing more than 10 % tumor cells.

The various potential failure modes tested in the robustness study for both the RNXtract and the MammaTyper assays affecting binding and washing steps and PCR parameters did not significantly alter the output. For the extraction experiments this involved adequate yields of RNA at concentrations which did not differ significantly from standard conditions except when the protein digestion step was omitted. For the MammaTyper the method failed upon omission of the reverse transcription step. As expected, the failure was detected by out-of-range median Cq values of the positive control. For the other two failure modes there was perfect agreement with respect to single markers.

### LoB, LoD, LoQ, linearity

All blank measurements showed no amplification signal up to 40 cycles. The LoQ was equal to the LoD for all six assays on IVT-RNA as well as FFPE derived RNA and both platforms Versant kPCR and LightCycler 480 instrument II. Data for the other metrics are summarized in Table 3 for experiments run on the two different qPCR systems for the FFPE RNA master sample and IVT-RNA (positive control) respectively. All assays were linear at least up to 32.33 and 33.56 Cqs for the two qPCR platforms tested. Amplification efficiencies ranged from 99 to 109 %. The lower (Cq) border of the analytical measurement range (AMR) for IVT-RNA on the LightCycler platform was limited by the fixed instrument baseline settings of the test, whereas for the FFPE master-sample the lower (Cq) border of the dilution curve reflected the expression of the target genes which in turn was restricted by the actual concentration of RNA in the master-sample (96.7 ng/μl).

**Table 3:** Validation metrics of MammaTyper amplification for each different target sequence and sample type

Versant kPCR		*ERBB2*	*ESR1*	*PGR*	*MKI67*	*B2M*	*CALM2*
FFPE Master Sample	LoD [Cq]	34.36	35.54	32.33	35.66	35.80	34.78
	LoD [molecules]	10	5	87	7	7	8
	AMR [Cq]	21.05–34.36	19.13–35.54	21.13–32.33	23.97–35.66	16.78–35.80	23.90–34.78
	Efficiency [%]	106	105	106	101	100	111
	Linearity	0.999	0.999	0.995	0.999	0.999	0.998
IVT-RNA	LoD [Cq]	35.31	35.60	36.72	36.41	36.22	33.69
	LoD [molecules]	5	5	5	5	5	18
	AMR [Cq]	12.36–35.31	12.45–35.60	12.64–36.72	11.63–36.41	12.41–36.22	11.67–33.69
	Efficiency [%]	105	104	100	99	100	100
	Linearity	0.999	0.999	1.000	0.999	1.000	1.000
LightCycler 480 II		*ERBB2*	*ESR1*	*PGR*	*MKI67*	*B2M*	*CALM2*
FFPE Master Sample	LoD [Cq]	33.56	36.47	33.56	35.47	35.6	35.62
	LoD [molecules]	40	8	64	12	12	9
	AMR [Cq]	22.32–33.56	21.69–36.47	21.20–33.56	24.61–35.47	17.60–35.60	25.26–35.62
	Efficiency [%]	109	113	115	111	109	117
	Linearity	1.000	1.000	0.996	0.999	0.999	0.997
IVT-RNA	LoD [Cq]	34.64	35.21	35.44	34.91	34.97	36.51
	LoD [molecules]	18	18	18	18	18	5
	AMR [Cq]	17.34–34.64	18.52–35.21	17.56–35.44	19.27–34.91	17.9–34.97	18.87–36.51
	Efficiency [%]	106	109	101	103	107	103
	Linearity	1.000	0.999	1.000	0.999	0.999	1.000

### Precision

Precision was evaluated simultaneously for RNA extraction and for RT-qPCR with regard to various potential sources of analytical variability such as qPCR instrument, reagent lot/batch and site/operators. One hundred percent of all specimens and measurements yielded valid results according to assay specifications except in study 2 for one sample at site 1, for which extraction was repeated using a reserve FFPE section. As expected, no signals were detected for the negative controls up to a Cq value of 40. The calculated test results of the 16 specimens adequately represent a wide range of clinically encountered 40-ΔΔCq values of the individual genes, both positive and negative calls for individual markers.

Table 4 shows the estimated variance components (expressed as standard deviations) generated by analytical and pre-analytical factors as well as the overall variance, for studies run on LightCycler 480 instrument II platforms (Roche) (inter-run/site, intra-run data generated with 8 RNA-pools on 4 instruments, inter/within section/extraction data generated on 16 samples with 3 cycles as described for the Versant instrument). The assay specific inter-site standard deviation (SD) for the entire set of the test samples was between 0.14 and 0.20 Cqs for Study No1. In this study there were only minor differences in the variance reflecting different components of precision. This indicates that the actual triplicate measurement is the major source of analytical imprecision and further noise is introduced only to a minor degree when experiments are carried out on different days or different instruments. Moreover, variability seemed to be homogenously distributed across the measurements of the four target genes, indicating comparable assay performance.

**Table 4:** Analytical variation presented as standard deviations of the 40-ΔΔCq values (SD) with 95 % confidence intervals (CI) for LightCycler 480 instrument II, Roche

LightCycler 480 II		Study 1							Study 2	
		Inter-Site	Inter-Run	Inter-run				Intra-Run	Inter-Extraction	Intra-Extraction
				Device 1 (site 1)	Device 2 (site 2)	Device 3 (site 2)	Device 4 (site 3)			
*ERBB2*	SD	0.20	0.21	0.21	0.18	0.22	0.21	0.20	0.08	0.16
	95 % CI	0.15–0.30	0.19–0.23	n.a.	n.a.	n.a.	n.a.	n.a.	0.05–0.19	0.14–0.19
*ESR1*	SD	0.18	0.27	0.26	0.27	0.21	0.31	0.30	0.52	0.55
	95 % CI	0.13–0.29	0.24–0.30	n.a.	n.a.	n.a.	n.a.	n.a.	0.39–0.79	0.48–0.64
*PGR*	SD	0.14	0.15	0.14	0.12	0.19	0.16	0.22	0.07	0.17
	95 % CI	0.11–0.22	0.14–0.17	n.a.	n.a.	n.a.	n.a.	n.a.	0.04–0.26	0.15–0.20
*MKI67*	SD	0.20	0.18	0.18	0.14	0.15	0.23	0.20	0.05	0.17
	95 % CI	0.16–0.30	0.16–0.20	n.a.	n.a.	n.a.	n.a.	n.a.	0.03–0.68	0.15–0.20

All data using models without day (inter-day variance is completely explained (covered) by inter-run variance). The calculations of inter- and within-section SDs are derived from a single instrument (study 2), due to the use of pooled samples in study 1

The estimated parameter-specific and overall variance for studies on Versant qPCR Cycler platforms (Siemens) are depicted in Table 5. The estimates for assay specific inter-site SD for the entire set of the test samples ranged from 0.00 to 0.66 Cqs, whereby a value of 0 indicates that the higher-order variance component is completely covered by the lower order component. A small but noticeable gradual increase in variability was observed while progressing from lower to higher components of precision. The data presented in Tables 4 and 5, collectively reveal a characteristic difference in the noise involving all testing parameters between experiments conducted on different qPCR platforms. Interestingly, biological variability, represented by inter-extraction variance was lower or almost equal to the variability between different aliquots of the same RNA eluate (intra-extraction) for each one of the two instruments (study 2), but it was overall higher for the Versant instrument. Thus, the same sources of analytical variation tested under similar conditions but on different qPCR systems resulted in comparable but not identical variability of the measurements.

**Table 5:** Analytical variation presented as standard deviations of the 40-ΔΔCq values (SD) with 95 % confidence intervals (CI) for Versant kPCR Cycler, Siemens

Versant kPCR AD		Inter-Site	Inter- Extraction	Intra- Extraction	Inter-Run						Intra-Run	Inter-Lot
					Device 1 (Site 1)		Device 2 (Site 2)		Device 3 (Site 3)			
					Inter- Extraction	Intra- Extraction	Inter- Extraction	Intra- Extraction	Inter- Extraction	Intra- Extraction		
*ERBB2*	SD	0.48	0.21	0.41	0.35	0.31	0.15	0.39	0.00^b^	0.49	0.29	0.16
	95 % CI	0.38–0.67	0.16–0.31	0.38–0.44	n.a.	n.a.	n.a.	n.a.	n.a.	n.a.	n.a.	n.a.
*ESR1*	SD	0.66	0.22	0.42	0.35	0.27	0.00^b^	0.29	0.18	0.61	0.26	0.09
	95 % CI	0.53–0.90	0.16–0.33	0.39–0.46	n.a.	n.a.	n.a.	n.a.	n.a.	n.a.	n.a.	n.a.
*PGR*	SD	0.00^a^	0.14	0.28	0.07	0.25	0.10	0.25	0.23	0.33	0.25	0.14
	95 % CI	n.a.	0.11–0.20	0.26–0.30	n.a.	n.a.	n.a.	n.a.	n.a.	n.a.	n.a.	n.a.
*MKI67*	SD	0.40	0.14	0.31	0.19	0.27	0.10	0.26	0.08	0.38	0.27	0.12
	95 % CI	0.32–0.55	0.10–0.23	0.29–0.34	n.a.	n.a.	n.a.	n.a.	n.a.	n.a.	n.a.	n.a.

^a^inter-site variance is completely explained (covered) by inter-extraction variance

^b^inter-extraction variance is completely explained (covered) by intra-extraction variance

### Concordance of individual marker and subtype classifications

**Fig. 4:**
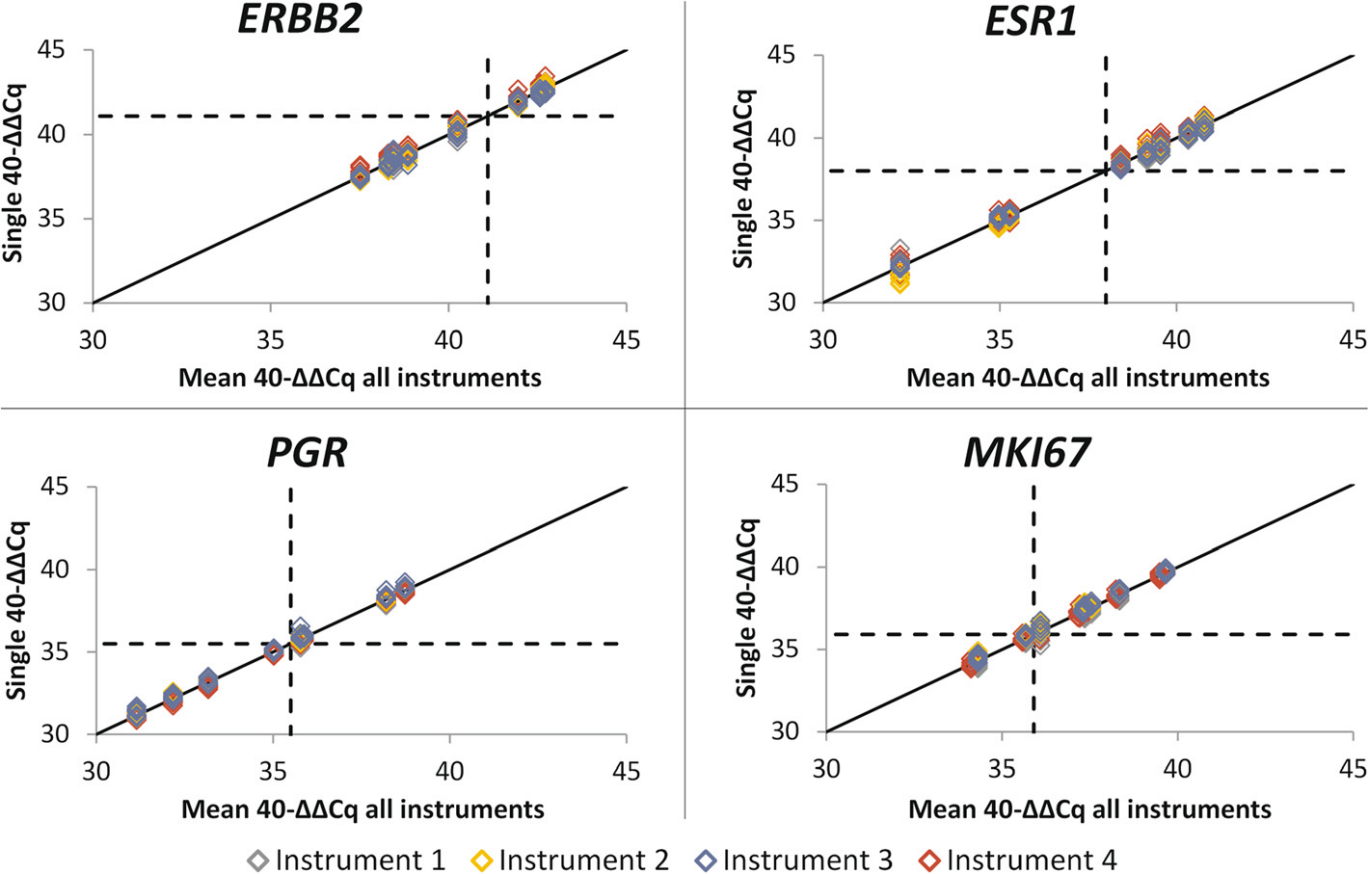
Correlation between single measurements and mean values over all measurements of 8 samples on 4 different LightCycler instruments (total of 24 measurements per sample)

The gold standard for assessing concordance was set by the most prevalent binary result across all measurements between sites after applying the respective cut-offs (positive versus negative). The average marker specific between-site and between-instrument concordance was over 97 % (Table 6) (over 94 % for subtypes) and it reflected excellent reproducibility of relative gene expression for the individual targets as shown in Fig. 4, for Study 1.

**Table 6:** Concordance of dichotomized test results between sites by qPCR system and between different qPCR platforms. The calculation of concordance between the two platforms is based on data from study 2

	*ERBB2*	*ESR1*	*PGR*	*MKI67*
Versant kPCR AD	98.1 %	94.7 %	96.3 %	95.1 %
LightCycler 480 II	100.0 %	100.0 %	98.4 %	94.3 %
Between platforms	100.00 %	96.9 %	97.2 %	98.6 %

## Discussion

Herein we have provided evidence that the quantification of *ERBB2*, *ESR1*, *PGR* and *MKI67* mRNA expression by using optimally standardized RT-qPCR assays is feasible, sensitive, specific and analytically precise.

The determination of individual markers and the combined assessment of breast cancer molecular subtypes remained unaffected by variations in tumor cell content or by the presence of adjacent DCIS. Robust and reproducible performance at different sites, including independent molecular pathology labs, where the methodology was introduced for the first time, resulted in highly concordant molecular subtyping of the tested samples.

Following the discovery of breast cancer molecular subtypes there have been multiple attempts to translate the recently acquired knowledge into clinically applicable gene-expression-based prognostic signatures and molecular classification tools [31]. This unprecedented explosion of diagnostic assays urged the Evaluation of Genomic Applications in Practice and Prevention (EGAPP) working group to articulate recommendations for the experimental development of molecular tests and the evaluation of supporting clinical utility data [24]. A major contribution has been the concept that clinical utility is verified not only upon convincing associations to clinical outcomes and treatments (clinical validity) but also through the demonstration of the test’s capacity to generate specific, sensitive and reproducible data under standard laboratory conditions (analytical validity) which is what we demonstrated herein for the MammaTyper IVD test.

Molecular tests currently marketed in breast cancer diagnostics are required to present published evidence of adequate analytical performance based on comprehensive technical studies [8, 32]. However, in practice, everyday prognostic stratification of cancer patients in breast oncology is based mainly on IHC, sparing proliferation gene signatures and molecular classifiers for a limited number of diagnostically challenging and equivocal cases [33]. Consequently, recent refinements in the criteria for analytical validity may not necessarily change the landscape for the large majority of routinely performed assessments of breast cancer biomarkers for prognosis and prediction. Of note, the identification of the target group of costly molecular tests still depends on less reproducible methods, such as IHC. Thus, reliable and accurate stratification of breast cancer patients in a practical and cost-effective way remains an important aim not only for molecular subtyping, but also for guiding the use of additional molecular assays which often involve shipment of tissues to distant, central laboratories.

In this study we have presented data on the MammaTyper RT-qPCR assay analytical performance according to predefined objectives and detailed planning of the validation process. The various analytical surveys, including those targeting the efficiency of the RNXtract RNA extraction method, were designed so as to satisfy guidelines issued by expert regulatory bodies such as the CLSI and MIQE to the highest feasible level. The combined extraction-amplification workflow was tested across a range of tumor samples that are similar to those routinely encountered in a clinical laboratory setting including both resection specimens and core needle biopsies. MammaTyper performed in a consistent and reproducible manner across that range in line with published characteristics of other RT-qPCR-based tests [32]. In addition, the assay was particularly stable not only within but also outside the specified RNA input limits as evidenced by concordant calls even upon failure of internal sample validity controls. Furthermore, consistent results were obtained: after reduction of input material to the half of the specified amount; by using alternative extraction methodologies or by deviating from standard conditions, lending additional support to the assay’s analytical robustness.

The precision studies were designed so as to differentiate between assay-specific variations and variation arising from regular tissue biological heterogeneity across multiple testing sites and 2 different qPCR platforms. The results of these studies illustrate that the MammaTyper assay is precise upon repetition over short or longer intervals of time. The various analytical parameters yielded only minor discrepancies in the test results.

The highest variance accounting for all measurements was 0.66 and 0.20 Cq values standard deviation for the Versant and the Roche cyclers, respectively. The variance is not identical between experiments conducted on the two different instruments and this affects all sources of analytical variation including tumor heterogeneity. This may indicate that the two platforms differ noticeably in terms of the generated analytical noise.

The molecular subtypes of breast cancer can be effectively approximated by surrogate definitions based on the expression status of only four markers. The extent to which this approach may be successful, directly relates to the performance of the methods employed at various levels of tumor classification and risk stratification. Herein we have demonstrated that the MammaTyper test can be reliably performed in a decentralized manner, yielding highly concordant results under different operating conditions. The level of accuracy obtained for single biomarker status is comparable to the best relevant numbers reported for HER2, ER and PR across various IHC studies [34–36]. Particularly for *MKI67* the level of reproducibility achieved herein by RT-qPCR is highly encouraging. Reliable and reproducible determination of tumor proliferation is at the moment difficult to achieve with IHC given the high discordance rates and difficulties in achieving guideline-driven improvements for Ki-67 [15].

The adherence to international guidelines, when available and the explicit validation of laboratory procedures are the cornerstones of establishing reliable and reproducible diagnostic assay performance. A recent study on the validation of predictive laboratory-developed IHC assays showed that almost half of the participants did not assess between-run precision and only less than half performed validations according to written protocols [37]. In a similar survey involving HER2, one in four responders failed to achieve the 95 % concordance rate, a target set by ASCO/CAP guidelines for positive and negative cases when comparing results between IHC and FISH with another IHC laboratory test for HER2 [38]. This alarming failure rate appears to be resistant to improvement according to a recent update of this study [39]. For ER on the other hand, low range expression or slightly different assay methods yielded different results, even between experienced observers or certified central laboratories [34, 40].

The higher reproducibility of RT-qPCR-based determination of the four breast cancer biomarkers which our results indicate should be interpreted while keeping in mind that this is a well designed analytical performance study which allows adequate control on devices and reagents and involves sites with more than average experience in molecular technologies. Therefore, regular rounds of quality control testing could be of assistance in achieving and maintaining high assay performance in local laboratories with variable background in molecular diagnostic applications. One of the major arguments against the potential use of RT-qPCR as an alternative to breast cancer biomarker detection by IHC has been the risk of false-negatives resulting from dilution of tumor cell RNA by other sources of RNA or the risk of false positives due to contamination by DCIS. Herein we demonstrate that the assessment of *ERBB2*, *ESR1*, *PGR*, and *MKI67* by MammaTyper in primary tumors remains unaffected by fluctuations in the TCC of FFPE specimens or by the presence of DCIS. Previous work showed that macrodissection does not alter the prognostic significance of *ERBB2* and *ESR1* mRNA [41] and that high concordance exists between paired invasive and *in situ* lesions when HER2 was assessed by IHC and FISH [42]. Our findings coupled with those of previous investigators may have direct implications on the handling of specimens for molecular diagnostics indicating that time-consuming performance of macrodissection could be spared at least for some markers.

The data presented herein for the RT-qPCR assay were obtained by processing of routine FFPE material with the RNXtract kit. The quality of the results therefore supports the use of the RNXtract paramagnetic bead assay as a highly efficient and specific method for RNA extraction intended for molecular diagnostic applications. In the pre-analytical factor study the RNXtract performed comparably to two other established RNA isolation methodologies. Furthermore, the high level of preferential RNA isolation achieved from a single 10 μm-thick section combined with the specificity of the MammaTyper assay make the use of DNA digestion unnecessary. This is of importance, as the effect of DNA digestion on gene expression signatures and their clinical interpretation has not been explored even for molecular assays already in routine use.

## Conclusions

During the past decades the ASCO/CAP guidelines have contributed substantially towards raising the level of standardization of IHC for HER2, ER and PR. Still, marker discordance rates between local and central testing often reported in the frame of modern, large clinical trials reveal the persistence of technical and interpretational limitations. Particularly for Ki-67, suboptimal analytical performance renders questionable the use of this marker for the selection of patients that are candidates for chemotherapy. From a technical perspective applying RT-qPCR for resolving the status of *ERBB2*, *ESR1*, *PGR*, and *MKI67* represents an efficient and reproducible alternative for decentralized routine assessment of breast cancer molecular subtypes. Herein we have provided evidence that the objective quantification of *ERBB2*, *ESR1*, *PGR* and *MKI67* mRNA expression by using optimally standardized RT-qPCR assays is feasible, sensitive, specific and analytically precise.

## Abbreviations

AMR, Analytical measureable range; ASCO/CAP, American Society of Clinical Oncology/College of American Pathologists; *B2M*, Beta 2 microglobulin gene/mRNA; *CALM2*, Calmodulin 2 gene/mRNA; CLSI, Clinical laboratory standards institute; Cq, Quantification cycle; DCIS, Ductal carcinoma in situ; ER, Estrogen receptor; *ERBB2*, Receptor tyrosine-protein kinase erbB-2 gene/mRNA; *ESR1*, Estrogen receptor gene/mRNA; FFPE, Formalin-fixed, paraffin-embedded; FISH, Fluorescence In Situ Hybridization; gDNA, Genomic DNA; GOI, Gene of interest; H&E, Hematoxylin and eosin; HER2, Receptor tyrosine-protein kinase erbB-2; IFU, Instructions for use; IHC, Immunohistochemistry; IVT, In-vitro transcribed; Ki-67, Marker of proliferation Ki-67; LoB, Limit of blank; LoD, Limit of detection; LoQ, Limit of quantification; MIQE, Minimum Information for Publication of Quantitative Real-Time PCR Experiments; *MKI67*, Marker of proliferation Ki-67 gene/mRNA; n.a., Not applicable; NTC, No template control; *PGR*, Progesterone receptor gene/mRNA; PR, Progesterone receptor; REF, Reference gene; RT-qPCR, Reverse transcription quantitative real time PCR; SD, Standard deviation; TCC, Tumor cell content

## Additional files

Additional file 1: A: Sample list, B: Assay cutoffs for single markers, C: Assay validity criteria, D: Robustness data. (XLSX 18 kb) Additional file 2: A: RNA and DNA concentrations in RNXtract eluates, B: Analyte specificity data, C: Graphical representation of variance components. (DOCX 125 kb)
