# Supplementary material for: Technical validation of an RT-qPCR in vitro diagnostic test system for the determination of breast cancer molecular subtypes by quantification of ERBB2, ESR1, PGR and MKI67 mRNA levels from formalin-fixed paraffin-embedded breast tumor specimens
Source: BMC Cancer. 2016 Jul 7;16:398. doi: 10.1186/s12885-016-2476-x (PMC4936300; doi:10.1186/s12885-016-2476-x)
Supplement: Additional file 2: — A: RNA and DNA concentrations in RNXtract eluates, B: Analyte specificity data, C: Graphical representation of variance components. (DOCX 125 kb) [file 12885_2016_2476_MOESM2_ESM.docx]

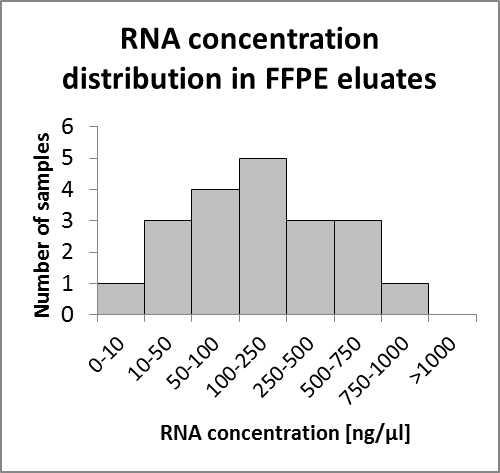

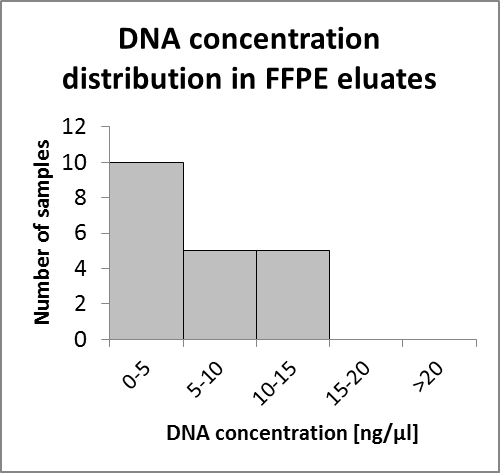


**A:** RNA and DNA concentration of 20 RNXtract® eluates.


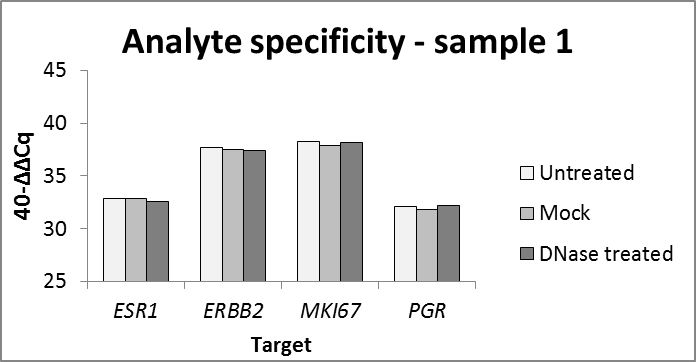

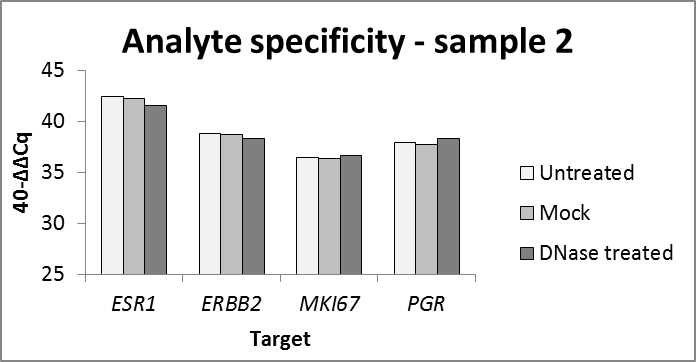

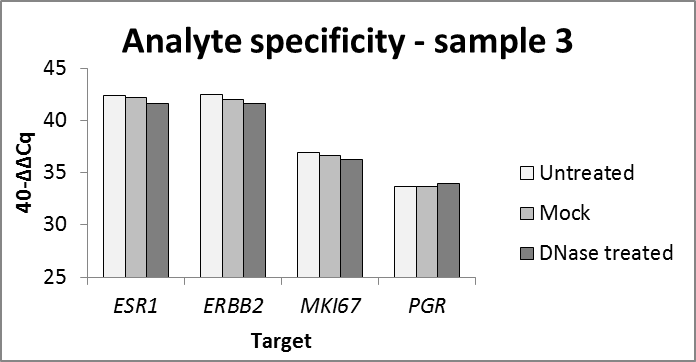


**B:** Analyte Specificity of MammaTyper tested on 3 independent RNXtract® eluates

**C:** Graphical representation of the different sources of variation (variance components) for the MammaTyper assay on the two instruments LightCycler 480 instrument II (Roche) and Versant kPCR (Siemens).
